# Intense ultraviolet–visible–infrared full-spectrum laser

**DOI:** 10.1038/s41377-023-01256-6

**Published:** 2023-08-22

**Authors:** Lihong Hong, Liqiang Liu, Yuanyuan Liu, Junyu Qian, Renyu Feng, Wenkai Li, Yanyan Li, Yujie Peng, Yuxin Leng, Ruxin Li, Zhi-Yuan Li

**Affiliations:** 1 School of Physics and Optoelectronics, South China University of Technology, Guangzhou, 510641 China; 2 State Key Laboratory of High Field Laser Physics and CAS Center for Excellence in Ultra-intense Laser Science, Shanghai Institute of Optics and Fine Mechanic Chinese Academy of Sciences, Shanghai, 201800 China

**Keywords:** Nonlinear optics, Ultrafast lasers

## Abstract

A high-brightness ultrabroadband supercontinuum white laser is desirable for various fields of modern science. Here, we present an intense ultraviolet-visible-infrared full-spectrum femtosecond laser source (with 300–5000 nm 25 dB bandwidth) with 0.54 mJ per pulse. The laser is obtained by sending a 3.9 μm, 3.3 mJ mid-infrared pump pulse into a cascaded architecture of gas-filled hollow-core fiber, a bare lithium niobate crystal plate, and a specially designed chirped periodically poled lithium niobate crystal, under the synergic action of second and third order nonlinearities such as high harmonic generation and self-phase modulation. This full-spectrum femtosecond laser source can provide a revolutionary tool for optical spectroscopy and find potential applications in physics, chemistry, biology, material science, industrial processing, and environment monitoring.

## Abstract (web summary)

The intense and flat ultraviolet-visible-infrared full-spectrum laser is a revolutionary tool that enables optical spectroscopy to simultaneously resolve ultrafast dynamics of multiple physical, chemical, and biological processes of a sample.

## Graphical abstract

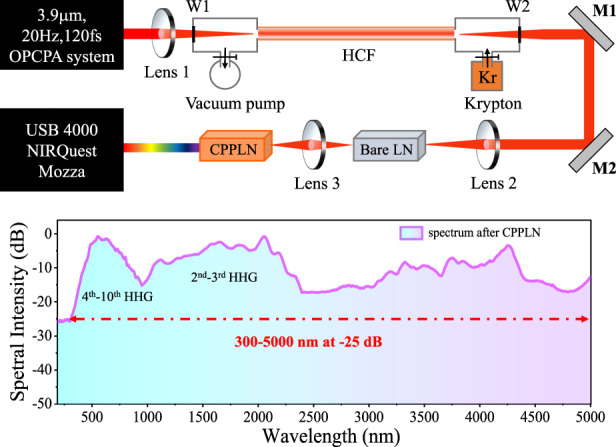

## Introduction

Optical spectroscopy from the ultraviolet (UV) across the visible (Vis) and into the infrared (IR) has proved to be a critical characteristic technique in probing the microscopic physical, chemical, and biological world^1^. Such a UV–Vis–IR full-spectrum optical spectroscopy is generally accomplished using a number of individual coherent light sources but is inevitably accompanied by complicated mechanical tuning^2^. Apparently, spectrally broad light sources that can cover multiple absorption bands and spectroscopic regimes are indispensable for simultaneously resolving multiple dynamic processes of gases, plasmas, liquids, and solids^3,4^. The collective action of laser technology and nonlinear optics has continuously pushed the spectral coverage to reach an unprecedented level. Yet, the direct generation of a high-brightness UV–Vis–IR full-spectrum white laser source is still an elusive technological capability.

Over the past decades, two different approaches have been developed for supercontinuum white laser generation. One approach is based on optical supercontinuum generation (SCG) technology by taking advantage of several third-order optical nonlinearities (3rd-NL) such as self-phase modulation (SPM) in microstructured optical fibers^5–8^, long-path gas-filled hollow-core fibers^9–11^, multiple thin silica plates^12,13^, or bulks^14,15^. Yet, the SCG spectral quality in terms of spectral bandwidth, spectral flatness, and pulse energy is inexorably subject to the tiny modal area or the complicated dispersion engineering. Another alternative way for building broadband laser sources is to manipulate various second-order nonlinearity (2nd-NL) effects such as second-harmonic generation (SHG), sum-frequency generation (SFG), third-harmonic generation (THG), and even high harmonic generation (HHG) in natural nonlinear crystals or specially designed microstructured nonlinear crystals like chirped periodically poled lithium niobate (CPPLN) via various phase matching or quasi-phase matching (QPM) schemes^16–23^. However, these 2nd-NL schemes are still poor in the performance of spectral and power scaling because of narrow pump laser bandwidth, limited QPM working bandwidth, and degraded energy conversion efficiency in higher-order harmonics.

In this work, we demonstrate an intense four-octave-spanning UV–Vis–IR full-spectrum laser source (300 nm to 5000 nm at −25 dB from the peak) coming from a cascaded HCF-LN-CPPLN optical module pumped by an intense mid-IR femtosecond pulse laser, which incorporates both 2nd-NL and 3rd-NL effects. First, we deliver a 3.3 mJ energy 3.9 μm pump laser into a gas-filled hollow-core fiber combined with a bare LN bulk crystal to create a 1.15 mJ one-octave-wide mid-IR laser supercontinuum covering 2500–5000 nm. Then, the pulse is delivered into a CPPLN crystal that exhibits multiple-order broadband reciprocal-lattice vector (RLV) bands of QPM allowing for simultaneous 2nd–10th HHG to occur efficiently. Moreover, the system involves considerable synergic action of 2nd-NL and 3rd-NL. Our demonstration illustrates the technological success of an innovative HCF-LN-CPPLN cascaded system for the implementation of an intense full-spectrum femtosecond laser, which would empower abundant opportunities for application in ultrafast and full-spectrum optical spectroscopy for physics, chemistry, materials science, and biology studies.

## Results

### Principle of full-spectrum laser generation and CPPLN design

**Fig. 1:**
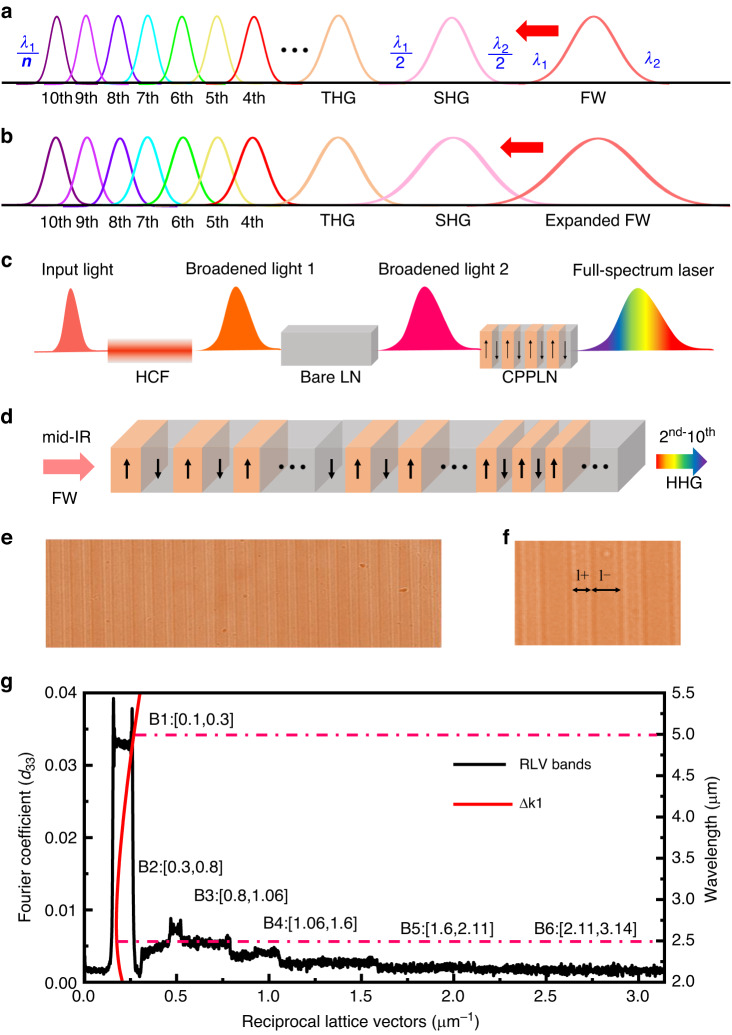
Principle for full-spectrum white laser generation. **a** Schematic diagram illustrating simultaneous broadband 2nd–10th HHG via cascaded 2nd-NL QPM up-conversion driven by a mid-IR pump femtosecond laser transporting in CPPLN. **b** Schematic diagram illustrating ultrabroadband 2nd–10th HHG supercontinuum white laser created under the pump of a high-peak-power mid-IR femtosecond laser via synergic action of 2nd-NL and 3rd-NL. **c** The mechanism of full-spectrum laser generation through a cascaded HCF-LN-CPPLN module via synergic 2nd-NL and 3rd-NL. **d** Schematic diagram for the structural geometry of the designed CPPLN crystal for HHG. **e** Optical microscopic image of the etched sample surface of a typical CPPLN structure. **f** Enlarged views of the positive and negative domains of the CPPLN sample. **g** Combined plots of the phase-mismatch curve for SHG process in a homogeneous LN crystal and the Fourier coefficient curves for the designed CPPLN sample in 2nd–10th HHG processes, which are divided into six QPM bands

The working principle of full-spectrum laser generation in our current system via synergic 2nd-NL HHG and 3rd-NL SPM effects is schematically shown in Fig. 1a, b. As depicted in Fig. 1a, suppose one can find an unusual but magnificent nonlinear crystal, for instance, a specially designed CPPLN crystal involving sufficient broad QPM bands, then simultaneous processes of broadband 2nd–10th HHG can be triggered via delivering an intense femtosecond fundamental-wave (FW) pump pulse laser with a certain bandwidth. However, the narrow bandwidth nature of the pump laser would result in significant discontinuities and gaps between harmonic output spectrum, especially among FW, SHG, and THG, if only 2nd-NL effects take action, even with high efficiency. It is natural to pose the question of what condition is needed to meet to generate a truly supercontinuum laser via HHG. We present a detailed description of the boundary condition required for interconnecting each harmonic band in Supplementary Note 1: Table S1. It can be seen that the minimum (*λ*_1_) and maximum (*λ*_2_) values of FW spectral band should obey a harsh condition that ${\lambda }_{2}\ge 2{\lambda }_{1}$ for generating multioctave full-spectrum femtosecond laser via HHG. At this one-octave-bandwidth pump prerequisite, we take the pump bandwidth ranging 2500–5000 nm as an example (see more details in Supplementary Note 1: Table S2). One can clearly find that the remaining FW pump and the generated 2nd–10th HHG signals are possible to preliminarily interconnect each other with certain spectral overlap in the case that the second precondition is satisfied: The CPPLN crystal has a sufficiently large operation bandwidth via a series of relevant QPM bands to drive high-efficiency HHG processes.

On the other hand, the 3rd-NL spectral broadening would be naturally driven by large peak power during high-efficiency 2nd-NL interactions occurring in the CPPLN sample. Specifically, the bandwidth of the pump laser can be expanded considerably, which creates and drives more frequency components to take part in the 2nd-NL up-conversion process. Thus, naturally the HHG signals will benefit from broader up-conversion bandwidth. Moreover, these high-peak-power HHG pulses would experience their own 3rd-NL SPM broadening effect, which help to generate a broader bandwidth for the 2nd-NL three-wave mixing processes involved within HHG and further expand the spectral bandwidth and smooth the spectral profile. Obviously, such a synergic 2nd-NL and 3rd-NL mechanism offers the potential to reach a high level supercontinuum white laser with flatter and smoother spectral shape due to the overlap of multiple spectrally broadened harmonics, as depicted in Fig. 1b. Foreseeably, under the pump of a high-peak-power mid-IR femtosecond laser exceeding one-octave bandwidth (${\lambda }_{2}\ge 2{\lambda }_{1}$) upon a CPPLN sample with high HHG conversion efficiency, the synergic action of 2nd-NL and 3rd-NL effects would expand the spectra of mid-IR pump laser to the short-wavelength region with a very high efficiency, finally driving a multioctave supercontinuum white laser output. Yet, technically it is difficult and challenging to fulfill these two preconditions for producing a full-spectrum supercontinuum laser with spectral coverage from UV to mid-IR range.

In our experiment, we present an innovative HCF-LN-CPPLN architecture to satisfy the above two preconditions: *Condition 1*, an intense one-octave pump femtosecond laser, and *Condition 2*, a nonlinear crystal with extremely large frequency up-conversion bandwidth. As briefly illustrated in Fig. 1c, a noble gas-filled HCF is used to generate the first-stage spectral broadening using a high-pulse-energy spectrally narrow mid-IR femtosecond pump. The first-stage broadened spectrum is further expanded by a bare bulk LN crystal, which leads to emission of new frequencies at shorter and longer wavelengths than the first generated spectrum. Here, the formation of a bright and balanced mid-IR pump supercontinuum relies on the 3rd-NL SPM process that exists in the HCF-LN setup. Then, this ultra-broadband mid-IR femtosecond laser serves as the high-peak-power seed to input and pump the engineered CPPLN crystal and drive continuous broadband 2rd-NL HHG processes. Finally, a flat multioctave-spanning supercontinuum laser is output from the cascaded HCF-LN-CPPLN module.

We begin by describing the design of the CPPLN sample that enables 2nd–10th HHG via efficient multiple-band QPM processes (see *Materials and methods* for more details). The structural geometry of a CPPLN structure is schematically shown in Fig. 1d. The chirped poling period gradually decreases from 41 μm at the left-hand to 23.79 μm at the right-hand with an optimized chirp rate *D*_*g*_ of 5.5 μm^−2^. The sample has a dimension in length, width, and thickness of 20 mm × 6 mm × 2 mm, respectively. Microscopic images of the etched CPPLN structure, fabricated using an electric poling technique^19,21,24^, are displayed in Fig. 1e, f. The calculated second-order nonlinear susceptibility Fourier-transform spectrum of the sample alongside the phase mismatching curve for SHG (against the FW wavelength) is displayed in Fig. 1g. The spectrum exhibits six continuous RLV bands with considerable Fourier coefficients. They are located at the region [0.1, 0.3] for band B1, region [0.3, 0.8] for band B2, region [0.8, 1.06] for band B3, region [1.06, 1.6] for band B4, region [1.6, 2.1] for band B5, and region [2.1, 3.14] for band B6, all in units of μm^−1^. Band B1 has the highest strength in effective nonlinear coefficient ${\chi }_{eff}$ (${\chi }_{eff} \sim 0.035{d}_{33}$, with *d*_33_= 27.2 pm/V) and still has a sufficiently broad RLV bandwidth. The other five bands have relatively smaller strength (compared to the ${\chi }_{eff}$ value of B1 bands) but are still at a modestly high level (${\chi }_{eff} \sim 0.003-0.01{d}_{33}$), simultaneously having very broad RLV bandwidth. Note that even with such a relatively small strength ${\chi }_{eff}$, a greatly enhanced conversion efficiency can still be achieved due to the high-peak-power femtosecond pump laser, while keeping broadband QPM interactions. Taking the SHG process as an example, the phase mismatching is perfectly covered by band B1. This means that efficient continuous broadband SHG process is enabled by QPM over the entire band of the pump laser. Thus, the six sufficient ultrabroad QPM bands within the sample together will work well to facilitate high-efficiency ultrabroadband HHG nonlinear optical up-conversion against the ultrabroadband FW pump pulse via various cascaded three-wave mixing processes. We have made a detailed QPM analysis for the 2nd–10th HHG nonlinear processes to effectively evaluate the performance of this designed CPPLN sample (see Supplementary Note 2 for details).

### Realization of intense UV–Vis–IR full-spectrum laser

**Fig. 2:**
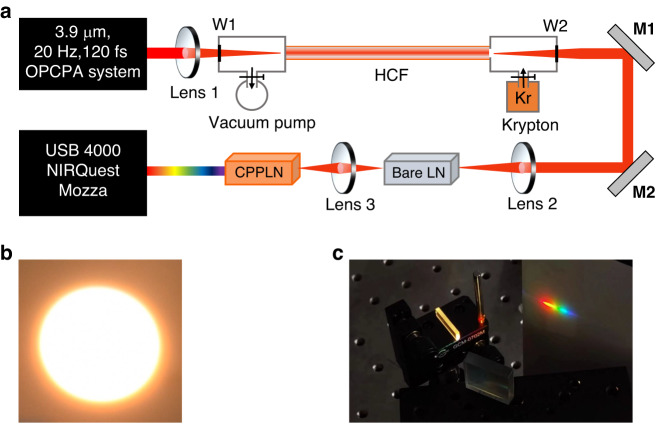
Experiment for the generation of full-spectrum laser from the cascaded HCF, bare LN and CPPLN module driven by intense mid-IR femtosecond pulse laser output from an OPCPA system. **a** Schematic diagram of the HCF-LN-CPPLN experimental setup. W, CaF_2_ window; M, mirror. **b** The bright white-light circular spot emitting from the CPPLN sample. **c** The 1st-order diffraction beam of (**b**), showing a colorful rainbow pattern from violet to red

The schematic of our experimental setup is shown in Fig. 2a. The full-spectrum white-light laser is achieved by launching an intense octave-spanning mid-IR laser that acquires from cascaded HCF and bare LN crystal module into a specially designed CPPLN sample to initiate high-efficiency 2nd-NL frequency up-conversion, which is pumped by a home-built 3.3 mJ 3.9 μm optical parametric chirped pulse amplification (OPCPA) system (for full details see *Materials and methods*). Figure 2b presents a bright and even dazzling supercontinuum white laser spot that is emitted from the end surface of CPPLN, which maintains well the original geometric shape and profile of the pump FW laser beam. Then, we use a regular grating to qualitatively assess the UV-Vis frequency components of the output white laser beam. As seen in Fig. 2c, the color of 1st-order HHG diffraction beam varies smoothly from purple to red. This naked-eye feature preliminarily illustrates that the output spectrum generated by the CPPLN sample contains complete visible light components.

**Fig. 3:**
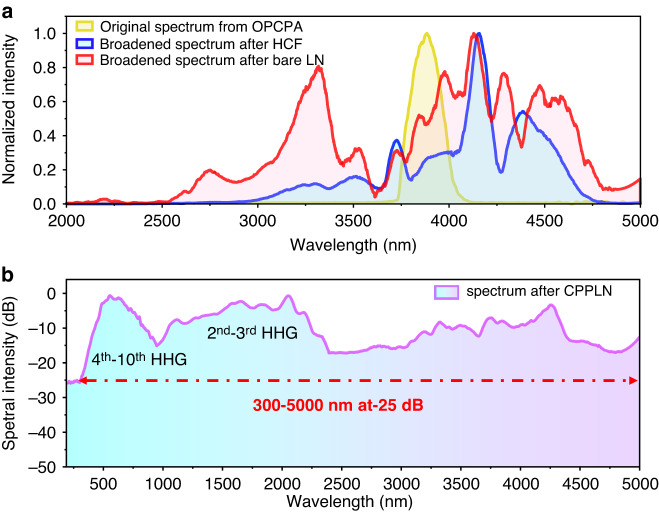
Measurement results of full-spectrum laser from the HCF-LN-CPPLN cascaded module. **a** The broadened spectra (normalized against the peak intensity) emitting from HCF (yellow), bare LN (blue), and the original output spectrum from OPCPA (red), respectively. **b** Normalized spectrum of the output full-spectrum laser signal generated by the HCF-LN-CPPLN module via the synergic action of 2nd-NL HHG and 3rd-NL SPM effects. The spectrum spans 300–5000 nm and encompasses a more than 4 octave bandwidth as estimated by a criterion of −25 dB. Note that the spectrum has been normalized against the maximum value

Further on, we collect each-stage laser output spectrum by three different optical spectrum analyzers for full UV–Vis–IR spectral characterization. As clearly shown in Fig. 3a, the spectrum of the 1.75 mJ pulse output from the HCF setup (the blue profile) is firstly effectively broadened, extending from 2.8 μm to 4.8 μm, at the Krypton pressure of 2.2 bar. It shows that the HCF device provides a significant spectral broadening due to the SPM effect. However, the short-wave wavelength edge at 2500–2800 nm and the long-wave wavelength edge at 4800–5000 nm are still vacant, which cannot meet the pump prerequisite (*Condition 1*) of full-spectrum laser generation (as described in Fig. 1a and b). Therefore, deliberate design is made so that a required broader spectrum covering 2500–5000 nm is achieved with 1.15 mJ pulse energy by using the next-stage SPM effect within LN bulk material (see the red profile in Fig. 3a). One can see that the frequency components among the entire mid-IR spectrum are redistributed with a greater balance, showing significantly broadened emission both in the short-wavelength part and long-wavelength one. This uniform spectral characteristic is desirable for enabling a multioctave supercontinuum laser with better flatness. We have systematically predicted the wavelength range of each harmonic via HHG across such a wide pump band, which has been summarized in Supplementary note 1: Table S2. We note that such an ideal pump light source can initially support the generation of a flat and smooth full-spectrum laser interconnecting the remaining pump FW and the 2nd–10th HHG output signals. As expected, Fig. 3b shows that the entire supercontinuum spectrum recorded from the CPPLN sample spans 300-5000 nm at 25 dB bandwidth, which demonstrates notable spectral flatness, smoothness, and continuity over the entire UV–Vis–IR supercontinuum bandwidth. Moreover, the output laser energy after CPPLN reaches quite a high level up to 0.54 mJ per pulse when subject to the input of the engineered broadband pump laser of 1.15 mJ per pulse in energy. One thing worth mentioning is that the maximum of the measured supercontinuum is limited to ≤5000 nm by our available spectrometers, whereas it is possible that the generated wavelength components go far beyond 5000 nm in our experiment.

In previous works, supercontinuum from photonic optical fiber yields more than three octaves in the spectral range 200–2500 nm, but only acquires a pulse energy at the very low picjoule level^7^. Supercontinuum from bulk materials can acquire very high pulse energy but have far smaller spectral bandwidth as limited by complicated dispersion-engineering^25^. Supercontinuum output with nearly seven octave bands is obtained by combining microstructure fiber and nonlinear crystal, but the spectral range of 340-40000 nm is calibrated at a much lower −70 dB level bandwidth and the single pulse energy is only 0.45 μJ^26^. It is fairly to say that the supercontinuum obtained by pure 3rd-NL effects in either microstructure fiber or bulk materials has many serious limitations. On the other hand, pure 2nd-NL HHG scheme via CPPLN can only support simultaneous 4th–8th HHG supercontinuum covering 400–1000 nm, whereas the spectral overlap among the first four harmonics is not possible because of the narrow-band pump feature^20^. Recently, we have successfully achieved a UV–Vis–IR supercontinuum white laser covering 350–2500 nm with a single pulse energy of 17 μJ from a single CPPLN crystal that enables simultaneous 2nd–10th HHG when it is pumped by a mid-infrared femtosecond laser with 45 μJ pulse energy via synergic 2nd-NL and 3rd-NL. However, due to the limited pump laser working bandwidth and QPM bandwidth, it is also very far from accomplishing the dream of the full-spectrum laser^23^.

The big success of our HCF-LN-CPPLN module mainly stems from the following three aspects, which have well cleaned up the shortcomings of individual 3rd-NL or 2nd-NL effects. First, this technology systematically takes advantage of the synergic action of 2nd-NL and 3nd-NL effects. The SPM effects from the HCF-LN setup have drastically improved the pump laser condition to better match with the versatile capability from CPPLN in terms of spectral intensity, bandwidth, and flatness. Second, the designed CPPLN sample supports a series of high-performance broad QPM bands that enable high-efficiency 2nd–10th HHG processes against intense one-octave pump laser via the flexible modulation of poling period structure, instead of the complicated linear dispersion engineering. Last but not least, CPPLN is a bulk material and naturally can endure a pump and output laser pulse energy many orders of magnitudes larger than the regular microstructured fiber optical systems only involving tiny modal area. Apparently, such a cascaded HCF-LN-CPPLN optical module enables access to a previously inaccessible level of intense full-spectrum laser output, which has not only an extremely large bandwidth (spanning 4 octaves), but also high-flatness spectral profile (from 300 to 5000 nm with a flatness better than 25 dB), and large pulse energy (0.54 mJ per pulse). It is expected the overall performance of full-spectrum laser in terms of spectral intensity, bandwidth, and flatness, and pulse energy can go to an even higher level if larger OPCPA output energy is used.

### Physics underlying up-conversion of one-octave pump laser

**Fig. 4:**
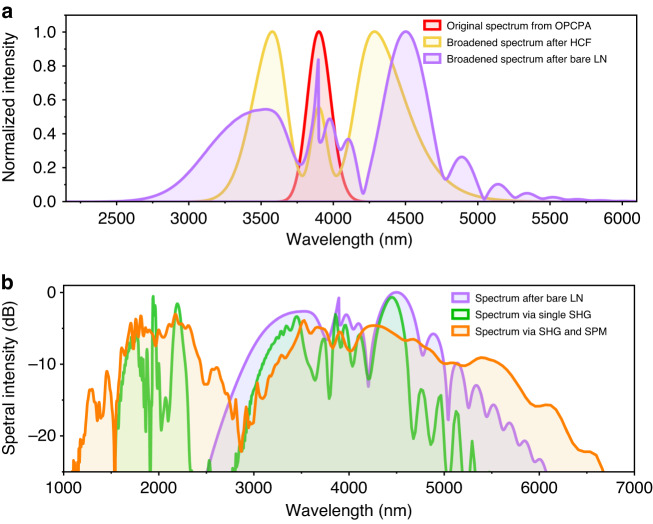
Numerical stimulation for the nonlinear interaction induced spectral evolution throughout the HCF-LN-CPPLN system. **a** Simulated output spectra (normalized against the peak intensity) from HCF (purple), bare LN (yellow), and the original pump mid-IR femtosecond laser spectrum from OPCPA (red), respectively. The spectrum broadens due to 3rd-NL SPM effects. **b** Simulated output spectra from the CPPLN sample pumped by the laser pulse emitted from the bare LN crystal (purple) with different nonlinear mechanisms. The green curve represents the output spectrum of the generated SHG signal and the remaining pump only via a single 2nd-NL effect, with a residual 455 nm spectral gap between them. The orange curve represents the interconnected output spectrum of the generated SHG signal and the remaining pump via the synergic action of 2nd-NL SHG and 3rd-NL SPM effects

According to the output spectrum displayed in Fig. 3b, one can find that the experimental spectrum between FW and SHG signal show higher continuity than the expected theoretical result only via HHG (as discussed in Supplementary Note 1: Table S2). Such a remarkable continuity among different HHG signals is good proof that the synergic action of 2nd-NL and 3rd-NL effects within CPPLN indeed occurs and enables to create a flatter and smoother supercontinuum spectral shape. To reveal the subtle details of physics, we theoretically model and numerically simulate the pump pulse propagation and nonlinear interaction within our system using parameters similar to our experiment and see how the originally far-away FW and SHG bands interconnect with each other. According to the analysis made in Supplementary Note 1: Table S2, the continuity between FW and SHG bands has imposed the most challenging burden in the success of UV–Vis–IR full-spectrum laser. We first set an initial mid-IR Gaussian pulse centered at 3.9 μm with a pulse duration of 120 fs and pulse energy of 3.3 mJ to act as the seed laser pulse. The simulated spectrum spans 520 nm. In the first step, we model the two-stage SPM spectral evolution within HCF-LN according to the simplified nonlinear Schrödinger equation (NLSE) to include the linear dispersion and SPM effects^27–29^. The calculated results are illustrated in Fig. 4a. One can see that the spectrum from the first-stage HCF has a significant primary broadening with a range of 3120–5120 nm, which is about four times the bandwidth of the input mid-IR femtosecond pulse laser. The next output spectrum after the second-stage LN bulk crystal spans more than one octave (from 2680 nm to 5580 nm), wherein exhibiting extensions to both the short- and long-wavelength sides of the input spectrum. This NLSE model in the HCF-LN system is used to examine the formation of a broad mid-IR supercontinuum in accordance with the experimental spectra shown in Fig. 3a. These numerical simulations show great consistency with experimental results, in terms of bandwidth, normalized efficiency, and broadening trend. The slight variations in the SPM-broadened spectra are attributed to the self-steepening and chaotic four-wave mixing and other adverse competitive processes that are ignored in our simulations.

On this basis of the simulated mid-IR supercontinuum laser source from the above 3rd-NL nonlinear broadening stage, we deliver it into the CPPLN sample to examine the output spectral profile. In this step, we focus on the phase-matched SHG nonlinear interactions to better understand the dynamics of SHG and FW. First, we evaluate the SHG process by only considering single 2nd-NL interaction. Here, we solve this separate 2nd-NL process by reference to the broadband nonlinear coupled wave theory^30,31^. The relevant result is shown in the green curve of Fig. 4b. The mid-IR supercontinuum pump laser from 2500 nm to 6068 nm at 25 dB bandwidth is effectively upconverted into a second-harmonic signal covering 1550–2345 nm. The remaining pump light shows a reduced spectral range from 2800 nm to 5321 nm with the energy consumption caused by the 2nd-NL effect. Obviously, the spectral bands of SHG and the remaining FW are not connected with each other, with a residual 455 nm spectral gap, which disagrees with the measured spectrum in Fig. 3b.

Things change dramatically when collective 2nd-NL and 3rd-NL effects are taken into account in the QPM SHG interaction simultaneously. We simulate this synergic ultrabroadband nonlinear interaction by solving a single envelope equation involving the two-NL power^32,33^ via the split-step Fourier method^34^. As seen in the orange curve of Fig. 4b, we note that the combined 2nd-NL and 3rd-NL effects generate a spectrally broadened second harmonic up to the range of 1100–2861 nm, much broader than the original value of 1550–2345 nm. Meanwhile, the spectral band of the remaining FW pump laser is extended from 2861 nm to 6660 nm, instead of the original band of 2800–5321 nm. This is mainly due to the fact that there is enough excess energy and power to support significant SPM broadening of the remaining pump laser. Obviously, the remaining FW and output SHG signal have their spectrum merge together and form a continuous spectrum spanning from 1100 nm to 6660 nm (as estimated by a criterion of −25 dB), which correctly captures many of the experimental features in terms of the spectral profile and extent. These simulation results clearly demonstrates that the spectral formation and evolution between SHG and FW observed in experiments result from a combination of 2nd-NL SHG and 3rd-NL SPM effects rather than only from a separate SHG process. It is worth noting that in the simulation, strong spectral signals are displayed at the band of 5000–6660 nm, which temporarily cannot be measured in our actual experiment due to the limitation of optical spectrometer. This also confirms the capacity of our experimental device to produce a wider supercontinuum spectrum extending to longer mid-IR band. In practice, nonlinear interactions within the CPPLN sample generate a series of HHG signals via a cascade of three-wave mixing processes. It can be fairly imagined that the full-spectrum supercontinuum envelope is governed by both high-efficiency HHG processes and SPM spectral broadening at multiple harmonics within the CPPLN sample, finally forming the entire four-octave bandwidth from the UV to mid-IR regions (see in Fig. 3b).

## Discussion

In summary, we have presented the successful realization of an intense four-octave UV–Vis–IR full-spectrum laser (25 dB bandwidth covering 300–5000 nm) with the energy of 0.54 mJ per pulse using a novel cascaded HCF-LN-CPPLN architecture. Under the pump of a 3.3 mJ with 3.9 μm mid-IR femtosecond pulse laser, the HCF-LN system can generate an intense one-octave bandwidth mid-IR laser pulse to serve as the secondary FW pump input into the CPPLN, while the CPPLN supports high-efficiency broadband HHG processes to further expand greatly the spectral bandwidth into UV–Vis–NIR. The high-efficiency synergic action of 2nd-NL and 3rd-NL effects is the secret for the success to create such a flat, smooth, and intense UV–Vis–IR full-spectrum white laser. Moreover, the HCF-LN-CPPLN system has the scaling-up capability to promote the performance of full-spectrum white laser in several crucial factors such as larger bandwidth, larger power energy, higher spectral brightness, and flatter spectral profile. Further works can be performed to expand the bandwidth into deep-UV and far-IR regions, while maintaining high spectral brightness and high spatiotemporal coherence. We expect that such an intense full-spectrum femtosecond laser can provide a revolutionary tool for optical spectroscopy and find potential applications in physics, chemistry, biology, materials science, information technology, industrial processing, and environment monitoring.

## Materials and methods

### CPPLN design

To achieve high-efficiency supercontinuum 2nd–10th HHG interactions, the relevant parameters of the CPPLN sample are the poling period needed to achieve phase-matching, and the effective strength of the interaction. The modulation period of the nonlinear susceptibility varying along the propagation direction is given by the formula Λ(*y*) = Λ_0_/[1 + (*D*_*g*_Λ_0_*y*/2*π*)], where Λ_0_ is the starting period length and *D*_*g*_ is the chirp rate. An effective nonlinear coefficient model is used to quantitatively evaluate the performance of various HHG three-wave mixing interactions in QPM CPPLN structures^35,36^. In our design, the central period is deliberately defined for the first-order QPM of SHG at the mid-IR wavelength of 3.9 μm. The sample has a total length of about 20 mm and the designed poling period ranges from 41 μm to 23.79 μm with the chirp rate *D*_*g*_ of 5.5 μm^−2^. Here, the feature of size asymmetry between the negative domain and the positive part in one period ensures high-performance odd and even order broad QPM bands (as described in Fig. 1g) with considerable effective nonlinear coefficients and provides the potential to support ultrabroadband HHG interactions with high conversion efficiency.

### Experimental setup

In the experimental setup shown in Fig. 2a, a home-built collinear OPCPA system provides a seed light source for the HCF-LF-CPPLN optical module. It creates a pump pulse centered at 3.9 μm with 3.3 mJ energy, a bandwidth of 400 nm, a pulse duration of 120 fs, and a repetition rate of 20 Hz^37^. Subsequently, this pump pulse is injected into a krypton gas-filled HCF with 1 mm inner-core diameter and 2.8 m length for the first-stage spectrum broadening. The focused spot diameter at the HCF inlet is controlled at 0.63 mm by an *f* = 800 mm CaF_2_ lens to get the maximum coupling efficiency^38^. In our experiment, we find that the optimal krypton pressure and input energy for nearly one-octave spectral expansion is 2.2 bar and 3.3 mJ with the compromise among spectral brightness and shape and thus we use these parameters. A 2-mm-thick CaF_2_ plate is placed at the output window to recompress the spectrally broadened pulse. A silver concave mirror is used to collimate the output beam for the next measurements. At this step, a high-peak-power mid-IR supercontinuum spanning from 2.8 to 4.8 μm with 1.75 mJ energy, 23 fs pulse duration (1.7 optical cycle), and 20 Hz repetition rate is delivered from the HCF setup^39,40^.

Next, the mid-IR supercontinuum laser beam is focused into a bare 20-mm-length LN bulk crystal through an uncoated *f* = 600 mm CaF_2_ lens (in a diameter of around 2 mm at the point of incidence) to initiate a further dramatic SPM spectral broadening. The spectral range is effectively expanded and covers 2500 nm to 5000 nm exceeding one-octave spanning, with an output energy of 1.15 mJ corresponding to a total transmission of 66 %. Afterwards, the emerging one-octave mid-IR supercontinuum laser is focused with an *f* = 100 mm CaF_2_ lens (to a beam waist of around 1 mm) into the designed *z*-cut CPPLN to trigger ultrabroadband 2nd-NL HHG frequency conversion, and finally generate a UV–Vis–IR full spectrum that covers four-octave bandwidth, 300–5000 nm at the −25 dB level.

### Spectral measurements

A combination of optical spectrum analyzers are used to measure the UV–Vis–IR spectra. The spectrum in the range between 200 nm and 1100 nm is measured with a UV–Vis spectrometer (Ocean Optics HR 4000, 200–1100 nm), the range between 900 nm and 2500 nm with an NIR512 spectrometer ((Ocean Optics NIR quest, 900–2500 nm), and the range between 1000 nm and 5000 nm with an acousto-optic dispersive filter (MOZZA, Fastlite Inc. 1000–5000 nm). We calibrate the three collected spectra by interpolating the data and stitching them together in a shared region where these three spectrometers are reasonably effective. Using this methodology, we finally obtain the spectra displayed in Fig. 3.

### Supplementary information

Supplementary Materials for Intense ultraviolet-visible-infrared full-spectrum laser

## Data Availability

The data that support the findings of this study are available from the corresponding author on reasonable request.
