# Supplementary material for: Intense ultraviolet–visible–infrared full-spectrum laser
Source: Light Sci Appl. 2023 Aug 22;12:199. doi: 10.1038/s41377-023-01256-6 (PMC10444876; doi:10.1038/s41377-023-01256-6)
Supplement: Supplementary file 1 — Supplementary Materials for Intense ultraviolet-visible-infrared full-spectrum laser [file 41377_2023_1256_MOESM1_ESM.docx]

Supplementary Materials for

Intense ultraviolet-visible-infrared full-spectrum laser

**Lihong Hong^1^, Liqiang Liu^1^, Yuanyuan Liu^1^, Junyu Qian^2^, Renyu Feng^2^, Wenkai Li^2^, Yanyan Li^2^, Yujie Peng^2^, Yuxin Leng^2^, Ruxin Li^2,*^, and Zhi-Yuan Li^1,*^**

^1^School of Physics and Optoelectronics, South China University of Technology, Guangzhou 510641, China

^2^State Key Laboratory of High Field Laser Physics and CAS Center for Excellence in Ultra-intense Laser Science, Shanghai Institute of Optics and Fine Mechanic Chinese Academy of Sciences, Shanghai 201800, China

*Correspondence should be addressed to Zhi-Yuan Li: [phzyli@scut.edu.cn](mailto:phzyli@scut.edu.cn); Ruxin Li: ruxinli@mail.shcnc.ac.cn.

**This file includes:**

Supplementary Text

Supplementary Fig. S1

Supplementary Table S1 to S3

**Supplementary Text**

**Supplementary Note 1 |** **Condition to generate supercontinuum laser via HHG.** As depicted in **Fig. S1a**, a prerequisite for the full-spectrum supercontinuum laser generation via HHG is that the harmonics should satisfy their respective spectral boundary conditions to connect with each other. Here, we set the minimum value of FW spectral band as $\lambda_{1}$, and the maximum one as $\lambda_{2}$. As displayed in **Supplementary Table S1**, to achieve the connection of FW and SHG, the maximum value of SHG spectral band needs to be greater than the minimum value of FW bandwidth, that is, $\lambda_{2}/2\geq\lambda_{1}$ so that $\lambda_{2}\geq2\lambda_{1}$. Similarly, the condition to realize the connection between SHG and THG is $\lambda_{2}\geq\frac{3\lambda_{1}}{2}$. By analogy, the conditions to achieve higher-order harmonics connected to each other (from THG to 10^th^ HHG signals) are $\lambda_{2}\geq\frac{4\lambda_{1}}{3}$, $\lambda_{2}\geq\frac{5\lambda_{1}}{4}$, $\lambda_{2}\geq\frac{6\lambda_{1}}{5}$, $\lambda_{2}\geq\frac{7\lambda_{1}}{6}$, $\lambda_{2}\geq\frac{8\lambda_{1}}{7}$, $\lambda_{2}\geq\frac{9\lambda_{1}}{8}$, and $\lambda_{2}\geq\frac{10\lambda_{1}}{9}$, respectively. Comparing the above conditions, one can find that a harsh condition of $\lambda_{2}\geq2\lambda_{1}$ need to be strictly obeyed for generating multioctave full-spectrum femtosecond laser via 2^nd^-10^th^ HHG. In this case, a high-peak power pump laser exceeding one-octave bandwidth would drive the generation of high-efficiency 2^nd^-10^th^ HHG within a specially designed CPPLN sample, wherein the spectral overlap between various harmonics increases gradually. Consequently, the remaining pump FW and 2^nd^-10^th^ HHG signals merge together and form a multioctave UV-Vis-IR full-spectrum supercontinuum laser.

In order to better understand this harsh condition of generating full-spectrum laser, we assume the FW band ranges from 2500 nm to 5000 nm and give a quantitative analysis of the corresponding bandwidth ranges of 2^nd^-10^th^ HHG. As shown in **Supplementary Table S2**, when illuminating the CPPLN sample by the FW laser covering 2500-5000 nm, the 2^nd^-10^th^ HHG output would encompass a sequence of broadband frequency domains of 1250-2500 nm, 833-1667 nm, 625-1250 nm, 500-1000 nm, 417-833 nm, 357-714 nm, 313-625 nm, 278-556 nm, and 250-500 nm. The first two spectral bands of FW and SHG would be connected at the wavelength of 2500 nm. Subsequently, the higher harmonics from SHG to 10^th^ HHG will go through the growing spectral overlap of 417 nm, 417 nm, 375 nm, 333 nm, 297 nm, 268 nm, 243 nm, and 222 nm. Evidently, such a one-octave-bandwidth pump laser source would be ideal for preliminarily meeting the demand of full-spectrum supercontinuum laser generation via HHG.

**Supplementary Table S1. The condition to generate supercontinuum laser via HHG. Here, $\lambda_{1}$ and $\lambda_{2}$ represent the minimum and maximum values of FW spectral band, respectively.**

| Harmonic order | Bandwidth range | Condition for harmonic connection |
| --- | --- | --- |
| FW | $\lambda_{1}\sim\lambda_{2}$ | $\lambda_{1}<\lambda_{2}$ |
| 2^nd^ HG | $\frac{\lambda_{1}}{2}\sim\frac{\lambda_{2}}{2}$ | $\frac{\lambda_{2}}{2}\geq\lambda_{1}\Rightarrow\lambda_{2}\geq2\lambda_{1}$ |
| 3^rd^ HG | $\frac{\lambda_{1}}{3}\sim\frac{\lambda_{2}}{3}$ | $\frac{\lambda_{2}}{3}\geq\frac{\lambda_{1}}{2}\Rightarrow\lambda_{2}\geq\frac{3\lambda_{1}}{2}$ |
| 4^th^ HG | $\frac{\lambda_{1}}{4}\sim\frac{\lambda_{2}}{4}$ | $\frac{\lambda_{2}}{4}\geq\frac{\lambda_{1}}{3}\Rightarrow\lambda_{2}\geq\frac{4\lambda_{1}}{3}$ |
| 5^th^ HG | $\frac{\lambda_{1}}{5}\sim\frac{\lambda_{2}}{5}$ | $\frac{\lambda_{2}}{5}\geq\frac{\lambda_{1}}{4}\Rightarrow\lambda_{2}\geq\frac{5\lambda_{1}}{4}$ |
| 6^th^ HG | $\frac{\lambda_{1}}{6}\sim\frac{\lambda_{2}}{6}$ | $\frac{\lambda_{2}}{6}\geq\frac{\lambda_{1}}{5}\Rightarrow\lambda_{2}\geq\frac{6\lambda_{1}}{5}$ |
| 7^th^ HG | $\frac{\lambda_{1}}{7}\sim\frac{\lambda_{2}}{7}$ | $\frac{\lambda_{2}}{7}\geq\frac{\lambda_{1}}{6}\Rightarrow\lambda_{2}\geq\frac{7\lambda_{1}}{6}$ |
| 8^th^ HG | $\frac{\lambda_{1}}{8}\sim\frac{\lambda_{2}}{8}$ | $\frac{\lambda_{2}}{8}\geq\frac{\lambda_{1}}{7}\Rightarrow\lambda_{2}\geq\frac{8\lambda_{1}}{7}$ |
| 9^th^ HG | $\frac{\lambda_{1}}{9}\sim\frac{\lambda_{2}}{9}$ | $\frac{\lambda_{2}}{9}\geq\frac{\lambda_{1}}{8}\Rightarrow\lambda_{2}\geq\frac{9\lambda_{1}}{8}$ |
| 10^th^ HG | $\frac{\lambda_{1}}{10}\sim\frac{\lambda_{2}}{10}$ | $\frac{\lambda_{2}}{10}\geq\frac{\lambda_{1}}{9}\Rightarrow\lambda_{2}\geq\frac{10\lambda_{1}}{9}$ |

**Supplementary Table S2. The achievable spectral range corresponding to the 2^nd^-10^th^ HHG emitted from the CPPLN sample and the contribution of QPM bands to each HHG under the pump of FW laser spanning from 2500 nm to 5000 nm.**

| Harmonic order | Bandwidth range (nm) | QPM band |
| --- | --- | --- |
| FW | 2500-5000 | / |
| 2^nd^ HG | 1250-2500 | B1 |
| 3^rd^ HG | 833-1667 | B1 |
| 4^th^ HG | 625-1250 | B1-B2 |
| 5^th^ HG | 500-1000 | B1-B3 |
| 6^th^ HG | 417-833 | B1-B5 |
| 7^th^ HG | 357-714 | B2-B6 |
| 8^th^ HG | 313-625 | B2-B6 |
| 9^th^ HG | 278-556 | B2-B6 |
| 10^th^HG | 250-500 | B3-B6 |

**Supplementary Note 2 | QPM analysis of CPPLN.** To clearly evaluate the availability of QPM mechanism of the CPPLN sample, we calculate the second-order nonlinear susceptibility Fourier spectrum curves as functions of the RLVs, namely $\chi_{eff}=\chi(G)=\int\chi^{(2)}(z)\exp(-iGz)\mathrm{d}z$. Numerically, the RLV composition of the structure can be calculated by applying the Fourier transform to the domain structure position function, which has a positive domain value of +1 and a negative domain value of −1. Collective plots of the Fourier coefficient curves in band B1-B6 alongside the phase-mismatch curves for 2^nd^-10^th^ HHG three-wave mixing processes are displayed in **Supplementary Fig. S1a-f**. The wave-vector mismatch Δ*k_i_* for 2^nd^-10^th^ HHG nonlinear frequency conversion processes in these figures is defined in **Supplementary Table S3**. As clearly plotted in **Supplementary Fig. S1a**, B1 band with the largest Fourier coefficient ($\chi_{eff}\sim0.035d_{33}$, with $d_{33}$= 27.2 pm/V ) successfully provides a good possibility to realize efficient SHG and THG processes through a complete coverage of the wave-vector mismatching $\Delta k_{1}$ and $\Delta k_{2}$ against a wide pump range from 2500 to 5000 nm. According to **Supplementary Fig. S1a and S1b**, the curves $\Delta k_{3}$ and $\Delta k_{4}$ are covered by bands B1 and B2, which are responsible for the 4^th^ HHG. In **Supplementary Fig. S1a-c**, we find that the curves $\Delta k_{5}$ and $\Delta k_{6}$ responsible for the 5^th^ HHG are mainly compensated by bands B2 and B3 with intermediate Fourier coefficients ($\chi_{eff}\sim0.005-0.01d_{33}$).

According to **Supplementary Fig. S1b-f**, the QPM responsible for the 6^th^ HHG via the $\Delta k_{7}$, $\Delta k_{8}$, and $\Delta k_{9}$ processes can be automatically achieved by the RLV bands of B2, B3, and B4 in the whole pump band, in which, band B2 with a relatively large Fourier coefficient acting the dominant role within the FW pump wavelength range of 3200-3800 nm. In the same manner, bands B2, B3, B4, B5, and B6 contribute towards the fulfillment of the QPM interactions responsible for the 7^th^ HHG via the required wave-vector processes $\Delta k_{10}$, $\Delta k_{11}$, and $\Delta k_{12}$. The phase mismatches $\Delta k_{13}$, $\Delta k_{14}$, $\Delta k_{15}$, and $\Delta k_{16}$ responsible for the 8^th^ HHG are also effectively compensated by bands B2, B3, B4, B5, and B6. Similarly, the 9^th^ HHG is enabled by four processes $\Delta k_{17}-\Delta k_{20}$ via the above five bands. The 10^th^ HHG is supported by five processes $\Delta k_{21}-\Delta k_{25}$ via B3-B6 bands. It can be concluded that the energies of the mid-IR femtosecond pulse laser within 2500-5000 nm can be effectively transferred to the SHG and THG signals in band B1 with a high conversion efficiency. Then, these two signals act as the starting point to ignite efficient HHG involving 4^th^-10^th^ harmonic outputs in the action of all QPM bands of B1-B6. That is to say, such a specially designed CPPLN sample with six continuous RLV bands is ideal for achieving simultaneous broadband 2^nd^-10^th^ HHG outputs against a high-peak-power pump femtosecond laser exceeding one-octave bandwidth, and therefore leading to the output of an ultrabroadband full-spectrum supercontinuum white laser.

**
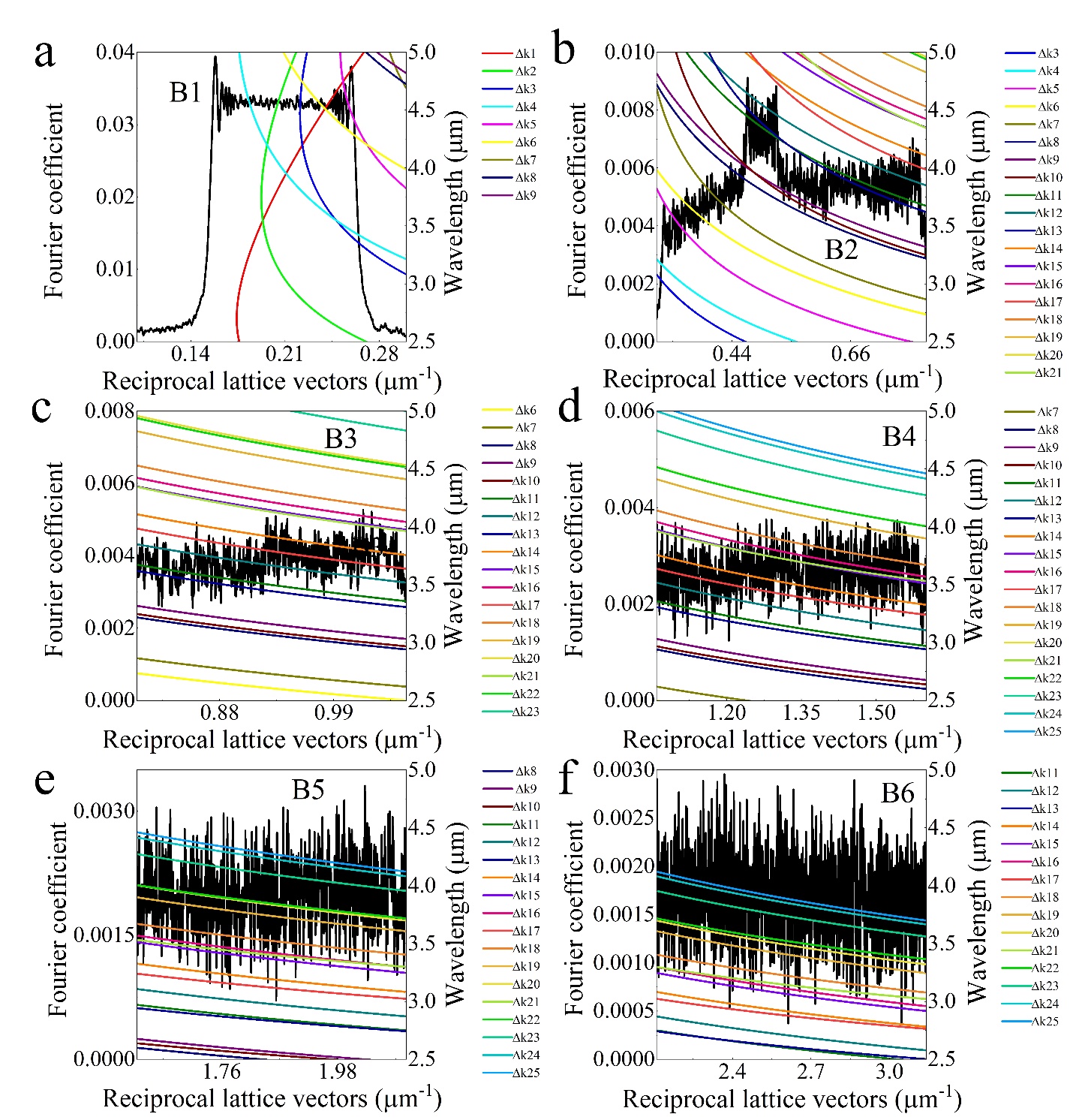
**

**Supplementary Fig. S1. Analysis of QPM schemes in CPPLN for 2^nd^-10^th^ HHG based on broadband three-wave mixing processes.** **a-f**, Collective plots of the Fourier coefficient curves in band B1-B6 offered by the CPPLN structure and the phase mismatch curves of various three-wave mixing processes in the CPPLN crystal. The efficient QPM three-wave mixing process arises when the overlap between the two curves occurs within the given mid-IR pump laser band.

**Supplementary Table S3. Contribution of QPM band and specific QPM three-wave mixing processes to various-order HHG.** $k_{i}$ **is the wave number of the *i*th HHG wave within a nonlinear crystal.**

| HHG | Phase mismatching in three-wave mixing processes | QPM band |
| --- | --- | --- |
| 2^nd^ HG | $\Delta k_{1}=k_{2}-2k_{1}$ | B1 |
| 3^rd^ HG | $\Delta k_{2}=k_{3}-k_{2}-k_{1}$ | B1 |
| 4^th^ HG | $\Delta k_{3}=k_{4}-k_{3}-k_{1}$, $\Delta k_{4}=k_{4}-2k_{2}$ | B1-B2 |
| 5^th^ HG | $\Delta k_{5}=k_{5}-k_{4}-k_{1}$, $\Delta k_{6}=k_{5}-k_{3}-k_{2}$ | B1-B3 |
| 6^th^ HG | $\Delta k_{7}=k_{6}-k_{5}-k_{1}$, $\Delta k_{8}=k_{6}-k_{4}-k_{2}$, $\Delta k_{9}=k_{6}-2k_{3}$ | B1-B5 |
| 7^th^ HG | $\Delta k_{10}=k_{7}-k_{6}-k_{1}$, $\Delta k_{11}=k_{7}-k_{5}-k_{2}$, $\Delta k_{12}=k_{7}-k_{4}-k_{3}$ | B2-B6 |
| 8^th^ HG | $\Delta k_{13}=k_{8}-k_{7}-k_{1}$, $\Delta k_{14}=k_{8}-k_{6}-k_{2}$, $\Delta k_{15}=k_{8}-k_{5}-k_{3}$, $\Delta k_{16}=k_{8}-2k_{4}$ | B2-B6 |
| 9^th^ HG | $\Delta k_{17}=k_{9}-k_{8}-k_{1}$,$\Delta k_{18}=k_{9}-k_{7}-k_{2}$,$\Delta k_{19}=k_{9}-k_{6}-k_{3}$,$\Delta k_{20}=k_{9}-k_{5}-k_{4}$ | B2-B6 |
| 10^th^ HG | $\Delta k_{21}=k_{10}-k_{9}-k_{1}$,$\Delta k_{22}=k_{10}-k_{8}-k_{2}$,$\Delta k_{23}=k_{10}-k_{7}-k_{3}$,  $\Delta k_{24}=k_{10}-k_{6}-k_{4}$, $\Delta k_{25}=k_{10}-2k_{5}$ | B3-B6 |
